# Basic Research of Material Properties of Mycelium-Based Composites

**DOI:** 10.3390/biomimetics7020051

**Published:** 2022-04-21

**Authors:** Hana Vašatko, Lukas Gosch, Julian Jauk, Milena Stavric

**Affiliations:** Faculty of Architecture, Institute of Architecture and Media, Graz University of Technology, 8010 Graz, Austria; lukas.gosch@tugraz.at (L.G.); julian.jauk@tugraz.at (J.J.); mstavric@tugraz.at (M.S.)

**Keywords:** mycelium, growth, bio-composites, mechanical properties, architecture, materials science

## Abstract

The subject of this research is growing mycelium-based composites and exploring their basic material properties. Since the building industry is responsible for a large amount of annual CO_2_ emissions, rethinking building materials is an important task for future practices. Using such composites is a carbon-neutral strategy that offers alternatives to conventional building materials. Yet, in order to become competitive, their basic research is still needed. In order to create mycelium-based composites, it was necessary to establish a sterile work environment and develop shaping procedures for objects on a scale of architectural building elements. The composite material exhibited qualities that make it suitable for compression-only structures, temporary assemblies, and acoustic and thermal insulation. The methodology includes evaluating several substrates, focused on beech sawdust, with two mycelium strains (*Pleurotus ostreatus* and *Ganoderma lucidum*), density calculations, compression tests, three-point flexural tests and capillary water absorption. The results of this study are presented through graphical and numerical values comparing material and mechanical properties. This study established a database for succeeding investigations and for defining the potentials and limitations of this material. Furthermore, future applications and relevant examinations have been addressed.

## 1. Introduction

Contemporary problems, such as rapid population growth, the increased demand for food and housing, freshwater scarcity and inadequate waste management, are all influenced by demographic, environmental and economic factors. The current linear economic model relies on the extraction, transformation and disposition of raw materials once their life cycle ends. Meanwhile, the concept of circular economy is defined by the reuse of materials, with radical changes in the production itself taking place, thus preventing the accumulation of waste [1].

An additional fact driving this research is that embodied carbon emissions from the manufacturing of building materials and the construction sector account for 38% of annual worldwide greenhouse gas emissions in the current world climate [2]. Out of the four raw material types currently extracted—minerals, ores, fossil fuels and biomass—the major portion, totalling approximately 40%, finds its use in the construction and housing sector [3]. If the European energy strategy with net-zero emissions is to be realised by 2050 [4], it is time to reconsider present design concepts as well as building elements and materials.

One way to accomplish this is by investigating new bio-based building materials. In the last decade, bio-fabrication gained significance in architecture and has become an integral part of sustainable building strategies. The production of mycelium-based composites is a low-energy and carbon-neutral process [5] that fits into the circular economy and sustainable building concepts. The utilisation of mycelium-based composites is wide in terms of scale, functionality and application, as several architects, designers and enthusiasts have begun to use them in their designs during the past decade. Furthermore, the composite has found its application as packaging material (*Ecovative*), acoustic insulation (*MOGU*) and as temporary objects exhibited in a larger scale, such as MycoTree [6] and the Growing Pavilion [7]. In comparison to building materials such as concrete or bricks, mycelium-based composites are a new term in architecture. Hence, there is still a high demand for basic research and testing in this respect. Several works have already presented some initial research by combining different substrates and mycelium strains and subsequently elaborating on some of their mechanical properties [8,9,10,11,12], and morphology and mechanics [13]. The work presented in this article extends the relevant references by providing an overview of the basic material properties in very specific material combinations, which include organic substrates, organic fibrous materials and inorganic materials. The introduction of inorganic and fibrous materials as substrates—such as clay, sand and soy silk fibres—contributes to the mentioned references. Since a wide variety of substrates and mycelium strains are present, as well as several decisive factors during the production process, the specification of the material properties is relevant. These preliminary experiments introduce the concept of growing mycelium-based lignocellulosic products, whose properties may subsequently be fine-tuned based on the material’s intended application. The overarching purpose of this research is fabricating heterogeneous composites with a defined material distribution, which will optimise the structural properties within one geometry. This study offers the initial point of evaluating material properties that will be used in these experiments.

Broadly speaking, mycelium is the vegetative part of mushrooms, which consists of branching hyphae. Mycelial growth can be described as a hyphal penetration of a substrate, which results in unifying it into one piece. A spore inoculated on a nutrient forms a tube which experiences exponential non-photosynthetic growth [14]. Three growth phases can be differentiated after inoculating a lignocellulosic substrate: (1) the lag phase (zero to little population growth, the mycelium cells get used to their new environment), (2) the exponential phase (if the conditions remain favourable, increase in biomass takes effect, as well as the cell number—this is the optimal period for mycelial growth and continuation of this for as long as possible is desirable) and (3) the stationary phase (the population growth returns to zero, the fungal biomass remains constant and some fungal cells may begin to perish) [15,16]. The oyster mushroom (*Pleurotus ostreatus*) mycelium was the most frequently used species for this research, as this very common mushroom type shows a high contamination resistance compared to other tested specimens and is capable of consuming a variety of lignocellulosic substrates. Its by-products, water, carbon dioxide, enzymes, alcohols and carbohydrates, serve as a nutritious foundation for other organisms in nature [17].

## 2. Materials and Methods

**Figure 1:**
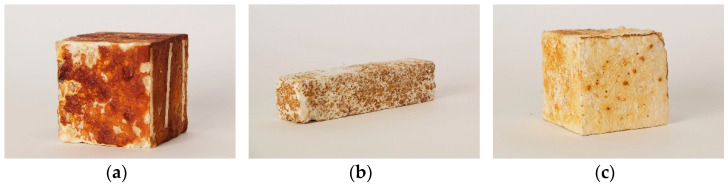
Samples composed of various materials: (**a**) cellulose pulp and clay; (**b**) beech sawdust and clay; (**c**) oak sawdust.

The methods of this research were carried out through material experiments and investigation of mechanical properties for an application as a building material. A series of material mixtures was created, as well as samples with different geometries for material testing experiments (Figure 1). The research presented here constitutes a database for subsequent investigations conducted by the authors, as well for the overall understanding of the potentials and limitations of the material. This initial study offers a comparison between different material qualities and their fine-tuned versions according to the function of their application.

Various organic substrates as well as inorganic additives were tested. However, beech sawdust was the initial substrate for assessing all tested material properties due to its availability in the local area, which minimises transportation and processing costs. Additionally, bleached cellulose pulp was a substrate option, as it achieved solid results and demonstrated no problems during inoculation and the growing phase, with very little to no contamination. Obtaining large quantities of cellulose in a desired form, however, proved to be challenging, leading to the choice of beech sawdust as the preferred substrate. The mycelium grain spawn used for this research was bought from a local vendor. Other substrates were also tested on individual samples, e.g., sand and clay, in order to increase the plasticity while comparing compressive strength.

All samples were given abbreviations within a naming system, which is used throughout this research: *Pleurotus ostreatus* mycelium (PO), *Ganoderma lucidum* mycelium (GL), beech sawdust (FS), oak sawdust (Q), bleached cellulose pulp (CS1), shredded cardboard (SC), shredded newspaper (SN), cotton fibres (CO), soy silk fibres (SF), wheat bran (WB), straw (ST), burlap (B), clay (C) and sand (S). The nomenclature works in a way that the abbreviation of the substrate is used firstly, followed by the abbreviation of the mycelium strain, and ending with a sample number. The abbreviations and the number sequences are connected with hyphens (e.g., FS-PO-01 is the first sample of beech sawdust inoculated with oyster mushroom mycelium).

The following sections provide a detailed description of the production procedure, initial substrate exploration, together with the mechanical material testing and evaluation of basic material properties. The initial substrate exploration includes substrate compatibility and investigating density. The mechanical material testing includes evaluation of compression strength and a three-point flexural test. Finally, determining the capillary water absorption coefficient was conducted.

### 2.1. Production Procedure of Mycelium-Based Composites

The starting point for producing mycelium-based composites was preparing the substrate by soaking it in distilled water for 24 h. Afterwards, the excess water was drained, and the moisture content (MC) of the substrate was measured. The substrate was then put in a polypropylene microfilter bag (PP50/SEU4/V40-51, *SacO2*) and sterilised in a pressure cooker at 121 °C for 45 min to eliminate any competing microorganisms that might hinder mycelial development. The moulds were made of perforated transparent foil with a thickness of 0.5 mm in order to produce samples for material testing, whose dimensions depended on the conducted method. Once the sterilised substrate cooled down to room temperature, mycelium grain spawn was homogeneously distributed in microfilter bags while working in a still-air box. The amount of mycelium used for inoculation was 10% of the weight of the sterilised substrate. The moulds were cleaned with rubbing alcohol (ethanol, 70% solution) and filled with the inoculated substrate by manual pressing. The microfilter bags containing the closed moulds were sealed and stored in an environment protected from light sources at temperatures ranging from 22 to 24 °C. The initial phase of development occurred in the moulds, followed by the second phase after unmoulding to achieve growth of the outer protective skin of the sample. The duration of each growth phase is defined in the succeeding sections. Once the mycelium fully colonised the substrate, dehydration was initiated in order to terminate the growth.

### 2.2. Initial Substrate Exploration

**Figure 2:**
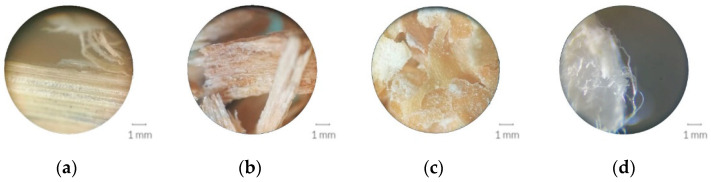
Substrate scale: (**a**) straw; (**b**) wood chips; (**c**) wheat bran; (**d**) bleached cellulose pulp.

Combinations of *Pleurotus ostreatus* and several substrates, including straw, beech sawdust, wheat bran and bleached cellulose pulp (Figure 2), were examined to evaluate breaking and shrinking, growth density, surface colour, and quality impression. The dimensions of the moulds were 10 × 10 × 2 cm. Four samples were prepared for each substrate type, with two of them incorporating a piece of burlap in the centre as additional organic reinforcement. The obtained burlap fabric was woven from jute and cut in squares with dimensions of 11 × 11 cm and had a fabric weight of 180 g/m^2^. The individual burlap pieces were larger than the moulds in order to visually assess the continuation of mycelial growth on the burlap. It was used simultaneously for nutrition, since jute fibres are composed of lignocellulose and for decreasing the shrinking of the composite. It was stiff and inelastic, and it had a moderate moisture regain [18]. Both burlap and the substrate were prepared according to the procedure described in the previous section. The moulds were not covered with a lid, as increased surface contact with oxygen accelerates mycelial growth [11].

**Figure 3:**
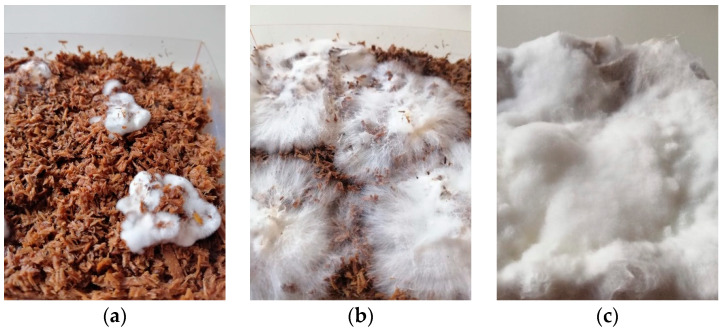
Close-up of the growth process of oyster mushroom mycelium on beech sawdust: (**a**) after three days; (**b**) after five days; (**c**) after 19 days.

In addition to successfully grown samples, a thorough growth documentation was obtained that demonstrates changes of the growth patterns and speed of the used mycelium strain as well as its preferred nutrition. The exponential growth phase [15,16] was visible after a couple of days only (Figure 3). Since the moulds were made of transparent plastic, the growth on the covered sides was noticeably reduced. After 20 days, the samples were unmoulded, flipped upside-down and placed back in the microfilter bag to achieve homogeneous surface growth on all sides. After the unmoulding, the second growth phase lasted for five days. Finally, the samples were dried over a heating source. The results of these samples are analysed in Section 3.1.

### 2.3. Material Testing

**Figure 4:**
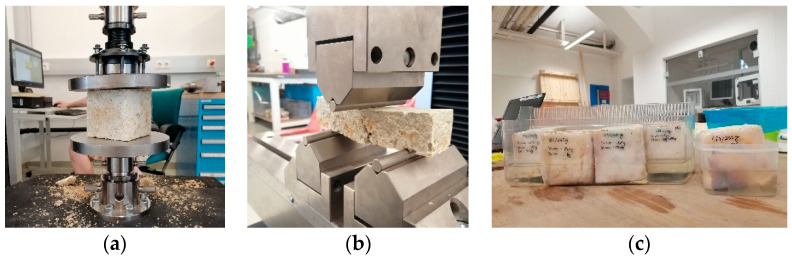
Setup for mechanical material testing and capillary water absorption: (**a**) compression strength; (**b**) three-point flexural test; (**c**) capillary water absorption.

Material testing included the evaluation of basic material properties and mechanical material testing. The former considered the calculation of density and capillary water absorption coefficient (Figure 4c), and the latter considered the compression strength and the three-point flexural test. The mechanical material testing was mostly executed at the Institute of Technology and Testing of Building Materials, Graz University of Technology (Figure 4a,b).

The samples used to estimate the density, as well as all subsequent material tests, were prepared according to the procedures outlined in Section 2.1. For this experiment, the growth duration lasted for 14 days, i.e., until the substrate was fully colonised by mycelium. The initial growth phase lasting seven days took place in plastic moulds and the second phase took place after unmoulding. Density was measured on samples grown in dimensions of 10 × 10 × 10 cm. A drying cabinet was used for sample dehydration. The temperature in the cabinet was set to 40 °C, and the drying process continued until the samples were completely dry. The documentation of weight loss and the calculation of the average water content is available in the supplementary documentation (Table S1). The method of determining the water absorption coefficient due to capillary action in hardened mortar [19] was used to determine the coefficient of water absorption of mycelium-based composites. The detailed description of the procedure and its results is provided in Section 3.5.

Both compression strength and the three-point flexural test were tested on a Shimadzu AG-X plus testing machine (Figure 4a,b). The standard used for these tests was EN 1015-11 [20], whereas the loading rate was 10 N/mm^2^/sec. Compressive strength was measured on the same samples that were used for density calculations (10 × 10 × 10 cm). The failure criterion of the compression strength tests was indicated by a drop of measured compressive force. This was caused by an abrupt deformation, which led to a loss of the overall integrity of the grown test samples. The samples for the three-point flexural test had dimensions of 4 × 4 × 16 cm and were placed on two linear bearings followed by a centrally applied linear load on top of those (Figure 4b). Regarding flexural tests, the failure criterion was indicated by a force drop right after achieving the peak value, which was caused by the occurrence of fractures in the sample. The graphical and numerical values of these experiments are presented in Section 3.3 and Section 3.4.

## 3. Results

### 3.1. Substrate Selection

**Figure 5:**
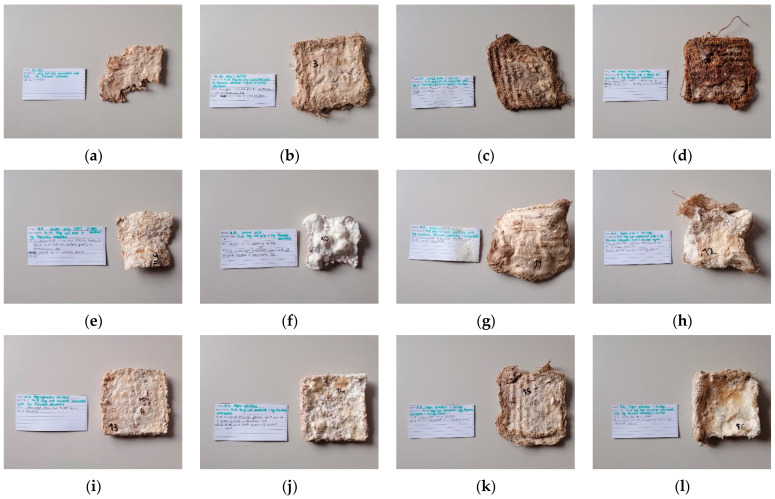
Samples of growth compatibility of *Pleurotus ostreatus* with straw, wheat bran, bleached cellulose pulp and beech sawdust: (**a**) ST-PO-01; (**b**) ST-B-PO-03; (**c**) WB-B-PO-03; (**d**) WB-B-PO-04; (**e**) CS1-PO-02; (**f**) CS1-PO-03; (**g**) CS1-B-PO-04; (**h**) CS1-B-PO-05; (**i**) FS-PO-20; (**j**) FS-PO-21; (**k**) FS-B-PO-22; (**l**) FS-B-PO-23.

The first set of samples contained straw as a substrate. It was chopped into single pieces no longer than 3 cm in length and hand-pressed into the moulds. The MC of the substrate was 60.23%. The straw fragments were visible after unmoulding and drying (Figure 5a,b). Because the substrate had not been thoroughly compressed prior to inoculation, the airiness in the material caused the samples to break. The shrinkage factor was negligible, yet the growth speed was sufficient, as the mycelium expanded across the surface in five days.

Using pure wheat bran as a substrate was not successful—the samples without burlap became contaminated, while the other two with the burlap piece only retained their form as the fabric kept them together. The MC of the substrate was 46.75%. The remaining samples were fragile. As observed on the dark surface of the samples, the mycelium was hardly visible (Figure 5c,d). However, using wheat bran as an additive would accelerate mycelium growth [21], and it will thus be used for this purpose in further experiments.

Pieces of bleached cellulose pulp (2–6 mm diameter, MC 64.17%) were dispersed in plastic moulds, and the mycelium grew entirely within. The substrate shrank by up to 40% after drying (Figure 5e,f), making it exceedingly unpredictable in cases where specified dimensions are to be achieved. The samples containing burlap shrank up to 5% (Figure 5g,h). Additionally, the samples became deformed during the drying process. To anticipate or, at the very least, to decrease the considerable shrinkage, the cellulosic substrate should be compressed firmly prior to inoculation. The samples had a white colour and a smooth surface.

Beech sawdust was pressed manually into the moulds after inoculation but became highly porous after the drying process. The MC of the substrate was 58%. Shrinkage was 10%, which is a reliable value for future use (Figure 5i–l). Similarly, as with all samples, the density of the substrate particles was important for the stiffness of the dried product. The outer layer of the samples was light brown, and the outer skin had not developed as uniformly as it did in the cellulose pulp samples.

### 3.2. Density

The samples were slightly distorted after the drying process, which happened due to the inconsistent pressure from the manual filling of the moulds and the standard shrinkage factor. They were measured from edge to edge and in the centre of each side to obtain an average side length, resulting in 24 measurements per sample. Consequently, the volume was calculated followed by the density (Table 1). In addition to beech sawdust, further density measurements of other substrates were carried out, such as bleached cellulose pulp, soy silk fibres mixed with beech sawdust, cotton, cardboard, beech sawdust mixed with sand, and beech sawdust mixed with clay.

### 3.3. Compression

#### 3.3.1. Compression—Beech Sawdust

**Figure 6:**
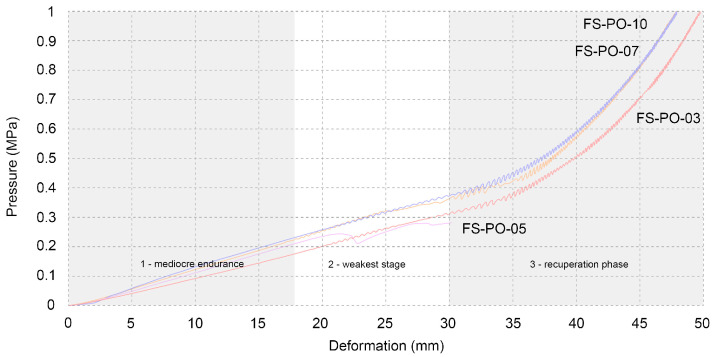
Compression test of beech sawdust samples.

The samples whose growth was terminated after 14 days (FS-PO-05 and FS-PO-10) showed similar compressive strength, as the ones that had three additional days to grow (FS-PO-03 and FS-PO-07) (Table 2). The curves on the graph are defined by three stages: the first showing mediocre endurance, the second being the weakest stage as the sample softens, and finally, the recuperation phase, in which the curve grows more steeply than it did previously (Figure 6). Results of this kind were to be expected, taking the porosity of the material into account.

#### 3.3.2. Compression—Various Samples

The various samples consisted of different combinations of organic and inorganic materials. A description of each sample is provided in the following text. The order of the samples is defined by their composition similarities.

FS-SF-PO-01—The sample consisted of horizontally stacked layers of soy silk fibres between layers of sawdust. Since the sample was loaded perpendicularly to the layers of soy silk fibres, it did not break apart as those made from sawdust had. The layers of fibres behaved as elastic springs, adding a certain flexibility to the sample. The ratio of soy fibres to sawdust is 1:1, inoculated with 20% *Pleurotus ostreatus*.

CO-PO-01—Since cotton consists mostly (88–97%) of cellulose [22], this type of nutrition was viable for mycelial growth. The fibres used for the sample had a significant length (5 cm), and the task of homogenising them with the mycelium grain spawn thus proved to be challenging. The performance of this sample can be ranked between the one with soy fibres and sawdust, and that with sawdust only. CO-PO-01 exhibited a similar quality that of the sample with soy fibres, since it was compacted after testing and did not really break.

SC-PO-01—The performance of this sample was high for withstanding compressive forces. Before inoculating with 10% *Pleurotus ostreatus*, the cardboard was soaked in hot water and later torn into small pieces—35 mm in length. The sample was found to have shrunk 11% when measured by the average side length and had a density of 0.42 g/cm^3^, which contributed to the best compression strength results (Table 3), as the cardboard was bound together tightly by the mycelium. 

FS-GL-01—This blend consisted of the same sawdust type as the ones in the previous section, but it was inoculated with *Ganoderma lucidum*. When compared to previous results, it did not perform as well.

**Figure 7:**
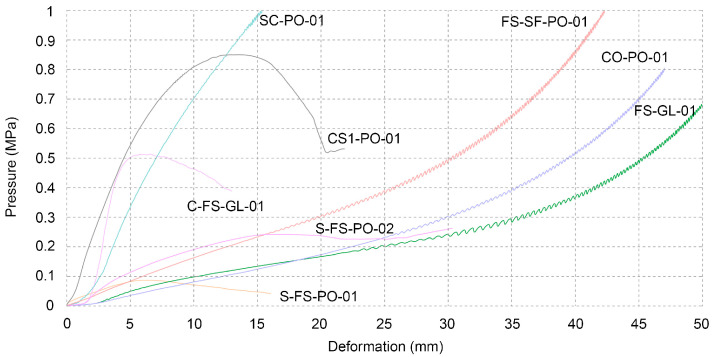
Compression test of various samples.

CS1-PO-01—The sample consisted of bleached cellulose pulp inoculated with 10% *Pleurotus ostreatus*. During the drying process, this sample shrunk significantly, as was expected after testing the substrate compatibility. The sides are reduced in size to 8.4 cm (from the original 10 cm per side). The sample thus has a density of 0.34 g/cm^3^, which is higher than that of the average sample made from beech sawdust (0.26 g/cm^3^). The sample was brittle, similar to the C-FS-GL-01, but withstood greater force (Figure 7). 

C-FS-GL-01—The mixture was made with one part of modelling clay to four parts of sawdust; the composite was inoculated with 10% of *Ganoderma lucidum* grain spawn. The organic portion was also influenced by the density of the clay—the goal was to introduce as much of the organic matter as possible in order to achieve a cohesive mycelial growth on the inside and as a result to enhance the properties of the composite. The sample showed similar brittleness as CS1-PO-01.

S-FS-PO-01 and S-FS-PO-02—The addition of sand to the mixture did not add to its mechanical strength. The two samples are differentiated by the amount of sand in the mixture: S-FS-PO-01 had equal quantities of sand and sawdust, while S-FS-PO-02 was based on a sand to sawdust volume ratio of 1:4.

### 3.4. Three-Point Flexural Test

#### 3.4.1. Three-Point Flexural Test—Beech Sawdust

**Figure 8:**
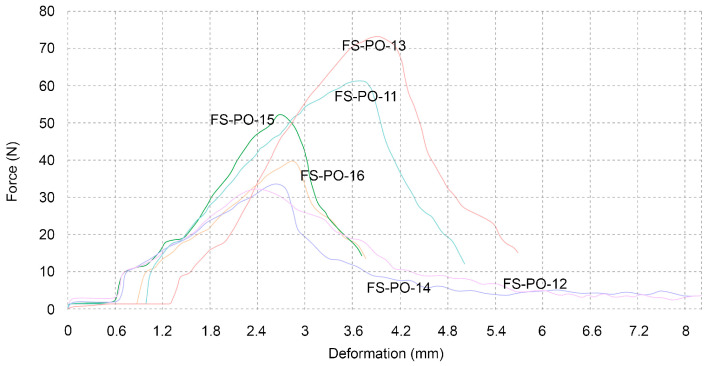
Three point flexural test of beech sawdust samples.

The first set of samples consisted of six beech sawdust samples. The numbers displayed in the table below show a wide dispersion of results (Table 4), despite them being simultaneously inoculated and incubated for the same period of time, under the same conditions. A conclusion of why the results vary so much cannot be drawn to one specific factor. However, when comparing the curve from the sample with the highest result, FS-PO-13 (Figure 8) is compared to an average result from the cellulose pulp samples, which are described in Section 3.4.2. A similar strain is not exhibited, whereby the sawdust samples can bear only half of the force that cellulose samples can.

#### 3.4.2. Three-Point Flexural Test—Various Samples

The following section describes individual composites tested in the same manner as the previous ones in Section 3.4.1. The first two samples, Q-GL-01 and FS-GL-19, were inoculated simultaneously with the same mycelium strain, *Ganoderma lucidum*, and grown under the same conditions for the same period of time. The substrate is the sole distinction between them, i.e., beech and oak sawdust. The sample inoculated with oak sawdust shows significantly better results (Table 5). It is important to note that the particles of oak sawdust were smaller (1–2 mm) compared to beech sawdust (about 3 mm). However, more samples are needed in order to make viable conclusions. This result difference will be further explored in order to relate the substrate type and mycelium strain to their specific mechanical properties. 

**Figure 9:**
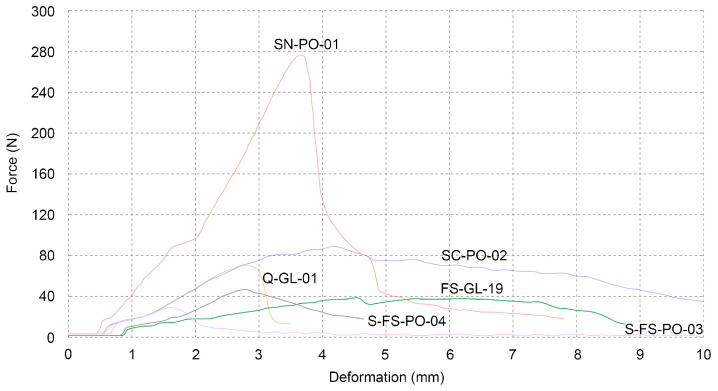
Three-point flexural test of various samples.

The sample SN-PO-01 showed the best results (Figure 9). This sample was prepared in a similar manner as the ones made with cardboard. Sheets of newspaper were soaked in water for 24 h, which were then torn into small pieces by hand. These samples performed five times better than the average values for beech sawdust. SC-PO-02, the sample containing shredded cardboard, showed lower results than the ones containing shredded newspaper, yet it performed better compared to the sawdust composites. The cardboard and newspaper pieces were approximately 15 mm long. 

The last two pieces containing sawdust and sand in different ratios, S-FS-PO-03 and S-FS-PO-04, showed no unexpected results. Adding sand did not improve flexural strength.

**Figure 10:**
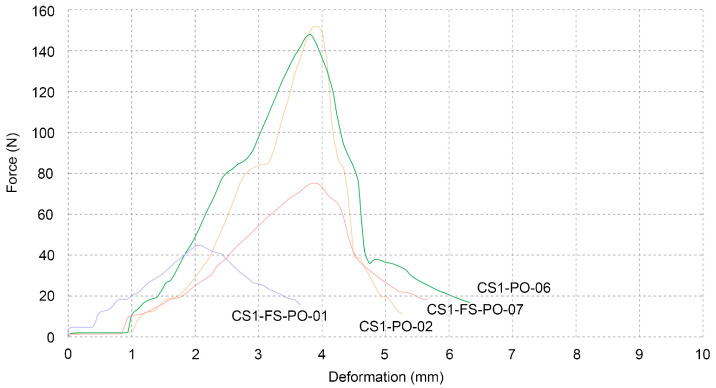
Three-point flexural test of bleached cellulose pulp compared to blend of bleached cellulose pulp with beech sawdust.

Samples CS1-PO-06 and CS1-PO-07 consisted entirely of bleached cellulose pulp and exhibited excellent mechanical properties when compared to the other tested samples. Another technique was explored by varying the ratios of the two basic components, cellulose pulp and beech sawdust. The first, CS1-FS-PO-01, was made up of 30% cellulose and 70% sawdust, while the second, CS1-FS-PO-02, was made up of 70% cellulose and 30% sawdust. These were used to investigate if adding another organic component improved or degraded the qualities that were being measured. Even though the sample with more cellulose pulp performed better, the 30% sawdust in the sample reduced its total performance when compared to the samples that solely contained cellulose. Adding 30% cellulose to the sample consisting mainly of sawdust did not drastically change the result when compared to the average of the sawdust samples (Table 6, Figure 10).

### 3.5. Capillary Water Absorption

The testing included several steps—firstly, the samples were completely dried out, and their surface was sealed with ethylene–vinyl acetate, which was applied using a standard glue gun. Once the dry weight of the samples was determined (M0), the container was filled with distilled water until the samples were immersed by 5 cm. The samples were placed in the filled container on a slant to ensure there was no air trapped beneath them, because of their uneven surfaces. The samples were weighed in a defined time range—after 10, 30, 60, 90 min, 2, 4, 8, and 24 h (Table 7). Five samples were examined, all of which had the same dimensions of 10 × 10 × 10 cm. Three of them were identical, consisting of beech sawdust inoculated with *Pleurotus ostreatus*, another with the same substrate but inoculated with *Ganoderma lucidum*, and an oak sawdust inoculated with *Ganoderma lucidum*.

For calculating the coefficient of water absorption, the following formula applies:

$$C=0.1\left(M4-M1\right)kg/\left(m^{2}\times min^{0.5}\right).$$

The last column of Table 7 shows a distinct dissociation of values; the samples inoculated with *Pleurotus ostreatus* and *Ganoderma lucidum*, with the latter showing a lower water absorption than the first sample group. These findings suggest that *Ganoderma lucidum* increases water repellence in samples inoculated with sawdust. *Pleurotus ostreatus* samples show water-repellent qualities, and *Ganoderma lucidum* samples show waterproof qualities.

## 4. Discussion

The substrates were chosen for their performance, but availability was also an important selection criterion. Out of the four initial materials that were evaluated, beech sawdust and cellulose pulp were considered to have potential for further research. Both exhibited adequate stiffness, growth density and a satisfactory quality impression. If compressed during inoculation, straw can also be considered a viable substrate, as it creates very lightweight and porous composites.

Density was measured on samples with initial dimensions of 10 × 10 × 10 cm, which was naturally decreased by mycelium digesting the substrate, compacting it and also by the drying process. In addition to these parameters, manually filling and compressing the moulds may have also influenced the density values of each sample.

The compression strength of beech sawdust composites had an average value of 2.49 MPa, with an interesting differentiation of samples whose growth lasted three days longer and exhibited higher values. Beech sawdust inoculated with *Ganoderma lucidum* did not perform as well as samples inoculated with *Pleurotus ostreatus*.

Three-point flexural tests were carried out on six beech sawdust samples with an average value of 0.11 N/mm^2^. Two samples inoculated with *Ganoderma lucidum* were compared, as the sample inoculated on oak sawdust showed better results. Shredded newspaper performed well as a substrate, with a value of 0.649 N/mm^2^. 

Cardboard and newspaper are the materials worth considering for future experiments. Both exhibit excellent compression strength values and the highest density from purely organic samples after mycelial growth. They are also usually discarded and can be recycled in this manner. However, additional research on these two materials is necessary, since they were tested on individual samples. Cellulose pulp exhibited excellent mechanical properties, but its dimensions after drying are not as predictable due to its high shrinkage. Using sand as an additive has shown stable results while documenting shrinkage, yet it is to be considered an improper additive, since it does not enhance the mechanical properties. Adding clay to the organic substrate was beneficial for the plasticity of the samples, and it will be researched further.

Finally, capillary water absorption was tested with two mycelium strains, *Pleurotus ostreatus* and *Ganoderma lucidum*. The samples inoculated with the former strain are water repellent, while the ones inoculated with the latter exhibit waterproof qualities. There was no difference between oak and beech sawdust in terms of water absorption.

The goal of this research was to evaluate various mycelium and lignocellulosic substrate combinations. This is important if mycelium-based composites are to be introduced into the building industry—consequently, the material samples were standardised and tested to be comparable with conventional building materials in terms of potential future applications. This series of tests was used to characterise and assess their properties. The data of this research will be used by the authors in order to further develop methods of evaluating properties of mycelium-based composites for specific applications, i.e., for researching heterogeneities in mycelium-based composites.

## 5. Conclusions

Mycelium-based composites exhibit structural properties that open up the possibility of their implementation in the building industry. Their applications include compression-only structures, temporary assemblies, art installations [23] and materials for acoustic and thermal insulation [24]. These have already been implemented as case studies and products developed in several companies. However, their application as a widely accepted alternative to some building components and commercialisation is yet to be seen. Moulds are a viable solution for shaping the material mixtures, yet the necessity of a sterile working environment, as well as the time mycelial growth takes, are somewhat limiting factors.

Within this research, it was possible to develop a fabrication process for mycelium-based composites on a scale of architectural elements similar to masonry units. Moreover, a sterile work environment was established, and a productive shaping method developed. The aim of this work was to gather data on several material properties and, as a result, to select the ones most suitable for composite materials depending on their application.

Another aspect that will be investigated is the correlation between growth time and the mechanical properties of mycelium-based composites, as seen in the results for different compression strength values of samples with a longer growth period. A series of samples will be made in which growth is interrupted in different samples and on numerous occasions, with a few days between each interruption. Another aspect that is planned to be looked into is the correlation between growth time, mechanical properties and weight loss of mycelium-based composites, as it has already been investigated on the decay of wood by brown-rot fungi [25]. This is important for defining the optimal growth advancements in the composite while retaining its maximal mechanical capacities.

**Figure 11:**
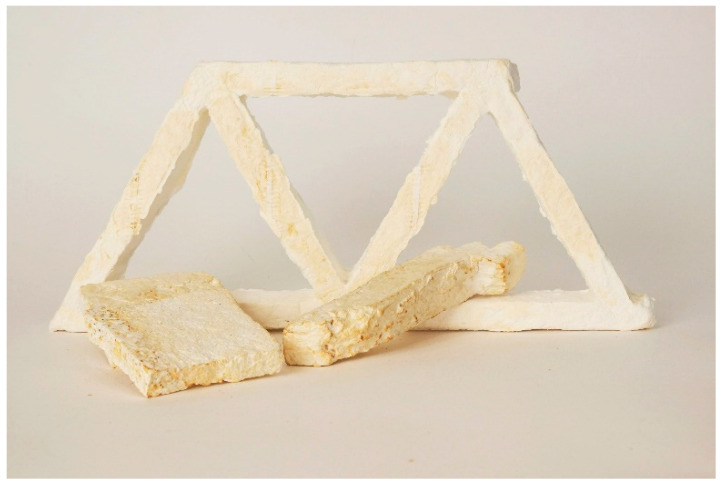
Truss structure made from cellulose pulp.

In addition to the fine-tuning of the composite by enhancing the desired material properties, the results presented here will be used for making heterogeneous material mixtures in complex geometries, including varying mechanical requirements. The major potential that is yet to be explored is controlled material distribution within a specific element. An initial experiment to test this hypothesis was conducted by creating a series of trusses consisting of different cellulose types, each exhibiting different mechanical properties (Figure 11). After thoroughly analysing the properties of homogeneous mixtures, a heterogeneous material distribution will be implemented.

In addition to exploring natural coatings to prevent the degradation of organic composites caused by moisture, the waterproof qualities of *Ganoderma lucidum* can be beneficial for its future use in mycelium-based composite materials. An experimental methodology has recently been developed in one study on the subject of biodegradability of mycelium-based composites based on soil burial tests [26]. These experimental methods are crucial if mycelium-based composites will be used in exterior applications. Using a certain mycelium strain that is more resistant to the water uptake is promising but still highly dependent on the type of substrate used [10]. Yet, their rapid biodegradability is one of their qualities that make the material appealing in terms of sustainability and waste management. Consequently, the focus of application of mycelium-based composites still remains in dry interior locations.

## Figures and Tables

**Table 1:** Density comparison (density of beech sawdust samples (FS) compared to various samples).

Name	Average Side (cm)	Volume (cm^3^)	Weight (g)	Density (g/cm^3^)
FS-PO-01	9.26	794.67	215.00	0.27
FS-PO-02	9.29	802.20	220.00	0.27
FS-PO-03	9.42	836.12	210.16	0.25
FS-PO-04	9.41	832.80	212.52	0.26
FS-PO-05	9.35	817.40	209.67	0.26
FS-PO-06	9.42	835.01	209.28	0.25
FS-PO-average	9.36	819.70	212.77	0.26
CS1-PO-01	8.74	668.01	225.00	0.34
FS-SF-PO-01	8.96	718.92	170.00	0.24
FS-GL-01	9.30	804.36	205.00	0.25
SC-PO-01	8.93	710.93	297.00	0.42
S-FS-PO-01	9.73	919.75	215.00	0.23
S-FS-PO-02	9.47	848.38	429.00	0.51
CO-PO-01	8.82	685.35	151.00	0.22

**Table 2:** Compression test of beech sawdust samples.

Name	Maximum Force (N)	Maximum Stress (MPa)
FS-PO-03	43,096.14	4.310
FS-PO-05	2822.56	0.280
FS-PO-07	42,275.38	4.230
FS-PO-10	11,166.73	1.120
average	24,840.20	2.490

**Table 3:** Compression test of various samples.

Name	Maximum Force (N)	Maximum Stress (MPa)
FS-SF-PO-01	15,737.45	1.990
C-FS-GL-01	4622.05	0.510
CS1-PO-01	6429.34	0.850
FS-GL-01	6607.95	0.760
SC-PO-01	20,975.92	2.650
S-FS-PO-02	2314.20	0.260
S-FS-PO-01	692.27	0.090
CO-PO-01	6224.60	0.800

**Table 4:** Three-point flexural test of beech sawdust samples.

Name	Maximum Force (N)	Maximum Stress (MPa)	Maximum Distance (mm)
FS-PO-11	61.11	0.14324	3.68
FS-PO-12	32.54	0.07626	2.40
FS-PO-13	73.02	0.17114	3.88
FS-PO-14	33.35	0.07816	2.66
FS-PO-15	52.37	0.12275	2.67
FS-PO-16	39.59	0.09280	2.85
average	48.66	0.11406	3.02

**Table 5:** Three-point flexural test of various samples.

Name	Maximum Force (N)	Maximum Stress (N/mm^2^)	Maximum Distance (mm)
Q-GL-01	70.38	0.16496	2.84
FS-GL-19	38.39	0.08997	4.52
SN-PO-01	276.95	0.64909	3.65
SC-PO-02	89.49	0.20973	4.23
S-FS-PO-03	29.18	0.06840	1.64
S-FS-PO-04	46.05	0.10792	2.74

**Table 6:** Three-point flexural test of bleached cellulose pulp compared to blend of bleached cellulose pulp with beech sawdust.

Name	Maximum Force (N)	Maximum Stress (N/mm^2^)	Maximum Distance (mm)
CS1-PO-06	147.95	0.34675	3.83
CS1-PO-07	151.95	0.35614	3.92
average	149.95	0.35145	3.88
CS1-FS-PO-01	44.87	0.10516	2.09
CS1-FS-PO-02	75.17	0.17617	3.92
average	60.02	0.14067	3.01

**Table 7:** Weight after certain periods of time. A water absorption coefficient greater than 2 is classified as strongly absorbent, while less than 2 is classified as water resistant, less than 0.5 is classified as water repellent, and less than 0.001 is classified as waterproof.

Name	M0: Weight Initial (g)	M1: Weight after 10 min (g)	M2: Weight after 30 min (g)	M3: Weight after 60 min (g)	M4: Weight after 90 min (g)	M5: Weight after 2 h (g)	M6: Weight after 4 h (g)	M7: Weight after 8 h (g)	M8: Weight after 24 h (g)	M8-M1	M4-M1	C (kg/(m^2^ × min⁰⁵))
FS-PO-17	285	291	299	313	325	338	393	472	565	274	34	0.0034
FS-PO-18	280	286	292	302	308	311	327	254	423	137	22	0.0022
FS-PO-19	273	281	289	304	311	318	346	399	505	224	30	0.0030
Q-GL-02	240	241	244	247	248	249	258	290	577	336	7	0.0007
FS-GL-20	233	233	234	237	239	244	272	355	546	313	6	0.0006

## Data Availability

Not applicable.

## Supplementary Materials

The following supporting information can be downloaded at: https://www.mdpi.com/article/10.3390/biomimetics7020051/s1, Table S1: Measurements during the drying process and average water content calculation.
