# Supplementary material for: Basic Research of Material Properties of Mycelium-Based Composites
Source: Biomimetics (Basel). 2022 Apr 21;7(2):51. doi: 10.3390/biomimetics7020051 (PMC9150003; doi:10.3390/biomimetics7020051)
Supplement: Supplementary file 1 [file biomimetics-07-00051-s001.zip › biomimetics-1660699-supplementary.pdf]

Table S1. Measurements during the drying process and average water content calculation.

1	description container	symbol M_2 [g]	FS-PO-03	FS-PO-04	FS-PO-05	FS-PO-06	FS-PO-07	FS-PO-10
2	container mass + moist measurement sample	$M_1 + M_2$ [g]	287.00	300.00	304.00	248.00	257.00	278.00
3	container mass + dry measurement sample	$M_{d1} + M_2$ [g]	210.18	212.54	209.75	209.28	218.72	217.96
4	second weighing container mass + dry measurement sample	$M_{di} + M_2$ [g]	210.16	212.52	209.67	209.28	218.68	217.90
5	weighing difference	3-4 [g]	0.02	0.02	0.08	0.00	0.04	0.06
6	[(3 - 4)/(3 - 1)]x100	[%]	0.01	0.01	0.04	0.00	0.02	0.03
7	water mass (2-4)	[g]	76.84	87.48	94.33	38.72	38.32	60.10
8	dry measurement sample mass (4-1)	M_3 [g]	210.16	212.16	209.67	209.28	218.68	217.90
9	water content (7/8)x100	[M-%]	36.56	41.16	44.99	18.50	17.52	27.58
10	average water content	[M-%]	40.91			21.20		
